# **Altered Bcl-2/Caspase signaling and hypoxia-induced apoptosis in primary human aniridia limbal stromal cells, in CoCl**_**2**_ **mediated hypoxic stress, *in vitro***

**DOI:** 10.1371/journal.pone.0328157

**Published:** 2025-07-10

**Authors:** Shanhe Liu, Shuailin Li, Shao-Lun Hsu, Fabian N. Fries, Zhen Li, Swarnali Kundu, Berthold Seitz, Maryam Amini, Shweta Suiwal, Julia Zimmermann, Simon Trusen, Tanja Stachon, Nóra Szentmáry

**Affiliations:** 1 Dr. Rolf M. Schwiete Center for Limbal Stem Cell and Congenital Aniridia Research, Saarland University, Homburg/Saar, Germany; 2 Department of Experimental Ophthalmology, Saarland University, Homburg/Saar, Germany; 3 Department of Ophthalmology, Saarland University Medical Center, Homburg/Saar, Germany; Faculty of Medicine of Tunis, TUNISIA

## Abstract

**Purpose:**

The aim of this study was to investigate apoptosis in primary aniridia limbal stromal cells (LSCs) and to assess changes in the expression of genes and proteins associated with the apoptotic pathway in response to cobalt chloride (CoCl_2_)-induced hypoxic stress, *in vitro*.

**Methods:**

Primary human limbal stromal cells were isolated from the limbal region of both aniridia (AN-LSCs; n = 8) and healthy (LSCs; n = 8) donors. The cells were treated with 0 µM, 50 µM, and 75 µM CoCl_2_ for 48 hours. Apoptosis in each group was assessed by Flow cytometry (FC). The expression levels of apoptosis-related genes, including CASP 3/7/8/9/10, BCL2, BID, BAX, CDKN1A (p21), CDKN1B (p27), TNFα, XIAP, and BIRC5 (Survivin), were measured by qPCR. Protein level of these markers was analyzed by FC. TNFα protein expression in the supernatant was quantified using ELISA.

**Results:**

Flow cytometry analysis revealed a significantly higher apoptosis rate in AN-LSCs compared to LSCs (p < 0.0001). In AN-LSCs, treatment with 75 µM CoCl₂ further increased the apoptosis rate compared to untreated AN-LSCs (p = 0.047). *BCL2* mRNA levels (p = 0.0291), Caspase-8 (p = 0.0341), Caspase-10 (p = 0.0085), Bcl-2 (p = 0.0014), XIAP (p = 0.0003) and Survivin (p = 0.0074) protein levels were significantly higher in LSCs than in AN-LSCs. Conversely, Caspase-3 (p = 0.0366), Caspase-9 (p = 0.0354), p21 (p = 0.0003), and p27 (p = 0.0164) protein levels were significantly higher in AN-LSCs than in LSCs. In LSCs, exposure to 75 µM CoCl₂ led to a reduction in *BCL2* mRNA (p = 0.0102) and protein levels (p = 0.0484), accompanied by an increase in *CDKN1B* mRNA level (p = 0.0265). In AN-LSCs, 75 µM CoCl₂ treatment resulted in a decrease in *CASP3* (p = 0.049), *CASP7* (p = 0.041) and *BCL2* (p = 0.0218) mRNA and Bcl-2 protein levels (p = 0.0405) and an increase of TNF-α protein levels in the cell culture supernatant (p = 0.0251).

**Conclusions:**

The apoptosis rate of LSCs from patients with congenital aniridia is higher than that of the control group, accompanied by alterations in multiple apoptosis-related markers. Additionally, CoCl₂-induced hypoxic stress further increases apoptosis in AN-LSCs and leads to changes in the expression of Caspase 3, Caspase 7, Bcl-2, and CDKN1B (p27). Further research is needed to elucidate the potential therapeutic targets in AAK, with the aim of preventing or slowing the progression of aniridia-associated keratopathy.

## Introduction

Congenital aniridia is a rare panocular condition characterized by the partial or complete absence of the iris (prevalence approximately 1:40,000–1:100,000), with common complications including aniridia-associated keratopathy (AAK), secondary glaucoma, juvenile cataract, macular and optic nerve head hypoplasia [1–3]. AAK is one of the leading causes of progressive vision loss in congenital aniridia. It is characterized by progressive limbal stem cell deficiency, corneal scarring, pannus formation and corneal neovascularization [3]. Abnormal corneal epithelial cell differentiation and migration, altered gene expression in aniridia limbal stromal cells, increased sensitivity to oxidative stress, impaired wound healing, and elevated apoptosis within the limbal niche may all contribute to the development and progression of AAK [4,5]. Interestingly, in cell culture, the viability, proliferation, and migration of aniridia-derived limbal stromal cells did not differ significantly from those of healthy controls [6,7].

Oxidative stress refers to the excessive production of highly active intracellular molecules such as reactive oxygen species (ROS) and an imbalance between oxidative and antioxidative pathways. This imbalance often occurs when the organism is exposed to environmental stressors or chemical agents and can lead to cellular damage. ROS not only contribute to oxidative damage but also function as molecular signals, activating various stress-sensitive intracellular pathways and initiating apoptosis [8]. One such chemical agent, Cobalt chloride (CoCl_2_), is widely used as a hypoxia mimetic and has been shown to induce ROS production, apoptosis, and transcriptional changes in genes such as hypoxia-inducible factor-1α (HIF-1α), p53, and p21 [9–11]. Studies have demonstrated that CoCl_2_ induces apoptosis in several types of stem cells [12,13]. Given that the cornea is continuously exposed to oxidative stress—particularly through UV light—ROS-related effects may influence cellular metabolism, migration, and overall corneal function [3,5,14]. In aniridia mouse models, the corneal epithelium has been shown to be particularly vulnerable to oxidative stress, resulting in increased apoptotic cell death [15,16].

Apoptosis is a form of programmed cell death through which cells undergo self-regulated elimination. This process is essential for maintaining tissue homeostasis and for the removal of damaged or abnormal cells. There are two main classical apoptotic pathways: the extrinsic and intrinsic pathways. The extrinsic pathway is initiated by the activation of cell surface death receptors (e.g., Fas, TNF receptors), which trigger apoptosis through the activation of effector caspases, such as Caspase-3. In contrast, the intrinsic pathway is activated by intracellular stressors—including DNA damage, hypoxia, and other cellular insults. This leads to the formation of apoptotic bodies, activation of effector caspases, and ultimately cell death. Although distinct, both pathways are interconnected and function in coordination, regulated by a complex network of signaling molecules to ensure that apoptosis occurs only under appropriate physiological or pathological conditions [17].

Stachon et al. identified alterations in apoptosis-related pathways in the protein profiles of conjunctival impression cytology samples from individuals with aniridia [18]. In addition, in aniridia limbal stromal cells, alterations of miRNA levels regulating apoptotic processes have been identified [19]. However, to the best of our knowledge, apoptotic signaling in limbal stromal cells (LSCs) from patients with congenital aniridia has not yet been investigated.

The purpose of this study was to explore apoptotic pathways in primary aniridia-derived LSCs under CoCl_2_-induced hypoxic stress *in vitro*. Our goal was to identify potential therapeutic targets within the apoptotic cascade that could be leveraged to favorably modulate the wound healing response.

## Materials and methods

### Ethics approval and consent to participate

This study was approved by the Ethics Committee of Saarland/ Germany (No. 178/22) and followed the rules of the Declasaion of Helsinki. All patients provided their consent in written form. The recruitment period of the prospective study started from 07.11.2022 to 15.12.2024.

### Cell culture

Aniridia limbal samples have been obtained from patients of the Department of Ophthalmology, Saarland University Medical Center, Homburg/Saar, Germany. Before surgery, all aniridia patients underwent a slit-lamp examination to grade AAK according to Lagali et al [20,21]. Control limbal biopsies were obtained from corneal donors of the LIONS Cornea Bank Saar-Lor-Lux, Trier/Westpfalz, Homburg/Saar, Germany.

1.5 mm limbal biopsies of 8 congenital aniridia eyes (age 32.50 ± 17.07 (2–50) years; 3 (37.5%) males) and 12 healthy donor eyes (age 81.17 ± 7.62 (64–90) years; 6 (50%) males) have been used for our experiments. Tables 1 and 2 provide details about all samples.

**Table 1:** Detailed information of the used healthy corneal donors.

Donor number	Gender	Age (years)
1	female	90
2	male	74
3	female	84
4	female	84
5	male	85
6	female	88
7	male	72
8	male	86
9	male	89
10	female	64
11	male	76
12	female	82
Total	6 (50%) male	81.17 ± 7.62 (64–90)

**Table 2:** Detailed information of the included congenital aniridia subjects. The aniridia-associated keratopathy (AAK) grade according to Lagali et al [23,24] is also included in the Table.

Genetic information	Grade	Gender	Age (years)
PAX6 missense mutation (c.1226-2A > G)	4	Male	43
PAX6 mutation (c.1191T(q227X))	3	Male	30
PAX6 missense mutation (c.607C > T)	4	Male	12
PAX6 missense mutation (c.1268A > T)	4	Female	50
PAX6 deletion (c.753_754delGC)	4	Female	46
PAX6 missense mutation (c.266A > C)	4	Female	36
PAX6 deletion (21q22.12q22. 236,472,360–39,889,694)x1	4	Female	2
PAX6 deletion (c.959_960delCA)	3	Female	41
Total	3 (25%); 4 (75%)	5 (62.5%) Female	32.50 ± 17.07 (2–50)

The extracted limbal biopsies were then placed in keratinocyte growth medium (KGM3, PromoCell) containing collagenase A (1 mg/ml) (Roche Diagnostic GmbH, Mannheim, Germany, No.10103578001) and were incubated at 37°C for 24 hours.

Isolation of primary LSCs has been performed as described by Chai et al. [22]. The suspensions were passed through 40 µm Flowmi cell strainers (No. H13680-0040, Bel-Art, Wayne, USA) into a 24-well plate to separate incompletely dissociated cells.

For LSCs cultures, the strainer was rinsed with 500 µl PBS followed by 500 µl of 0.05% trypsin-EDTA, and incubated for 5 minutes to disaggregate cell clusters. The enzyme activity was then halted by adding DMEM supplemented with 5% FCS. The resulting filtrate was centrifuged at 1500g for 5 minutes to pellet the LSCs, and the supernatant was discarded. The cells were then seeded in a 6-well plate using DMEM supplemented with 5% FCS. The cells were maintained at 37°C with 95% relative humidity and 5% CO_2_, with the medium being refreshed every 2–3 days until the LSCs reached confluence.

### CoCl_2_ treatment

LSCs were grown until reaching 80% confluence, before experiments. After 24 hours, the medium was substituted with DMEM solution that included CoCl_2_ (Sigma-Aldrich® GmbH, Geisenheim, Germany, No. 60818). This solution was left for 48 hours to create a hypoxic environment with CoCl_2_ doses of 0 µM, 50 µM, and 75 µM, respectively.

### Apoptosis

Apoptosis was detected using an Annexin V apoptosis detection kit (Invitrogen, Germany, No.2734867). Cells were trypsinized, centrifuged, and resuspended in 1xbinding buffer. The samples were incubated with 5 µL of Annexin V-APC at room temperature for 15 minutes, followed by the addition of 5 µL of propidine iodide (PI) and subsequent analysis by flow cytometry. Annexin V-APC and PI fluorescence were detected in the R660 nm and B585 nm channels, respectively. Non-apoptotic and apoptotic cells were distinguished based on Annexin V-APC and PI staining. Non-apoptotic cells showed no staining with either reagents. Apoptotic cells exhibited strong red (APC) fluorescence and low to moderate blue (PI) fluorescence, indicating early and late apoptosis. The increased permeability of late apoptotic cells to PI was due to compromised plasma membrane integrity.

The results were expressed as the percentage of apoptotic cells relative to the total cell population detected. We represented data as mean±SD from three independent experiments.

### RNA measurement

The RNA was extracted following the manufacturer’s instructions using the NORGEN RNA Purification Plus Micro Kit. The quantification of proteins and RNA was performed using the Bradford method and a UV/VIS spectrophotometer (Analytik Jena AG, Jena, Germany), respectively. The samples were then kept at a temperature of −80 °C. The synthesis of cDNA was performed using the OneTaq RT-PCR kit (New England Biolabs Inc. Ipswich, USA), 500 ng of total RNA per sample was used as the template. Synthesized cDNA was stored at −20 °C.

### Quantitative PCR

Table 3 provides a summary of the primers used for qPCR obtained from Qiagen GmbH (Hilden, Germany). As the manufacturer’s guidelines, the samples were tested using 1 µl of the appropriate primer solution, 5 µl of SYBR Green Mix (Vazyme, Nanjing, China), 3 µl of nuclease-free water, and 1 µl of cDNA. Each gene was measured in duplicate. The PCR Thermocycler QuantStudio 5 Real-Time PCR System, manufactured by ThermoFisher ScientificTM GmbH in Dreieich, Germany, was utilized. The amplification conditions consisted of a denaturation step at 95 °C for 10 seconds, followed by an annealing step at 60 °C for 30 seconds, and a final extension step at 95 °C for 15 seconds. This cycle was repeated 40 times. The cycling threshold (Ct) obtained from the measurements was adjusted by normalizing it to the average values of β-glucuronidase (GUSB) to calculate ΔCt (ΔCt = individual Ct value – Ct value of GUSB). ΔΔCt was then defined as the average ΔCt minus the individual ΔCt. The geometric mean was computed by taking the multiplicative change in ΔΔCt (geometric mean = 2 ^ΔΔCt^), which represents the difference between the Ct value of the target sample and the average Ct value of the reference gene in a squared multiple manner.

**Table 3:** Primer pairs used for qPCR.

Target cDNA	Referred as	Qiagen Cat. No	Amplicon size(bp)
Hs_CDKN1A_1_SG	CDKN1A	QT00062090	79
Hs_CDKN1B_2_SG	CDKN1B	QT00998445	146
Hs_CASP3_1_SG	CASP3	QT00023947	147
Hs_CASP7_1_SG	CASP7	QT00003549	149, 223, 256
Hs_CASP8_1_SG	CASP8	QT00052416	61
Hs_CASP9_1_SG	CASP9	QT00036267	102
Hs_CASP10_1_SG	CASP10	QT00002394	91
Hs_BRIC5_1_SG	BRIC5	QT00081186	101
Hs_BAX_1_SG	BAX	QT00031192	111
Hs_BID_1_SG	BID	QT00077833	98
Hs_BCL2_1_SG	BCL2	QT00025011	80
Hs_TNF_1_SG	TNF	QT00029162	98
Hs_XIAP_1_SG	XIAP	QT00042854	87
Hs_GUSB_1_SG	GUSB	QT00046046	96

### Flow cytometry

For LSCs and AN-LSCs, flow cytometry (FC) analysis was performed in three independent experiments. Cells were detached with trypsin without EDTA (Merck, USA, NO. T4424), counted, washed, and incubated with the appropriate antibodies. For staining with all markers, antibodies not conjugated to a fluorescent dye were used, and the secondary antibody (Antibody online, No. ABIN101988. Biotechne, No. F0118) was only used as a staining control. Stained cells were analysed immediately using a CytoFLEX flow cytometer (Beckman Coulter) and further analysed using CytExpert 2.6 software. Table 4 provides details about all antibodies.

**Table 4:** Antibodies used for flow cytometry.

Antibody	Referred as	Supplier	Catalog number	Source	Dilution
p21 Waf1/Cip1 (12D1) Rabbit mAb	p21	Cell Signaling	#2947	Rabbit	1:100
p27 Kip1 (SX53G8.5) Mouse mAb	p27	Cell Signaling	#3698	Mouse	1:100
Caspase-9 Antibody	Caspase-9	Cell Signaling	#9502	Rabbit	1:100
Caspase-3 Antibody	Caspase-3	Proteintech	#19677–1-AP	Rabbit	1:100
Cleaved Caspase-7 (Asp198) (D6H1) Rabbit mAb	Caspase-7	Cell signaling	#8438S	Rabbit	1:100
Cleaved Caspase-8 (Asp374) (E6H8S) Rabbit mAb	Caspase-8	Cell signaling	#98134S	Rabbit	1:100
Caspase-10 Polyclonal antibody	Caspase-10	Proteintech	#14311–1-AP	Rabbit	1:100
TNF-α (D1G2) Rabbit mAb	TNF-α	Cell signaling	#8184S	Rabbit	1:100
Bid Polyclonal antibody	Bid	Proteintech	#10988–1-AP	Rabbit	1:100
Bax (E4U1V) Rabbit mAb	Bax	Cell signaling	#41162S	Rabbit	1:100
Human Bcl-2 Polyclonal antibody	Bcl-2	Proteintech	#12789–1-AP	Rabbit	1:100
XIAP Polyclonal antibody	XIAP	Proteintech	#23453–1-AP	Rabbit	1:100
Survivin (71G4B7) Rabbit mAb	Survivin	Cell signaling	#2808S	Rabbit	1:100
Goat anti-Rabbit IgG (Heavy & Light Chain) Antibody (FITC) – Preadsorbed	–	Antibody online	ABIN101988	–	1: 500
IgG1 Antibody, anti-mouse, REAfinity	–	Miltenyi Biotec	REA1017	–	1:50

### ELISA

In this study, TNFα protein level in the supernatant of LSCs and AN-LSCs was also quantified using the DuoSet® ELISA Kit (R&D Systems, Bio-Techne, Minneapolis, USA, NO. DY210). The assay was conducted in accordance with the manufacturer’s protocol, and each measurement was performed in duplicate. Absorbance was recorded at 450 nm using the Tecan Infinite F50 Absorbance Microplate Reader (Tecan Group AG, Männedorf, Switzerland) to determine protein concentrations. To ensure accurate quantification, the obtained data were normalized against the total protein content.

### Statistical analysis

Statistical analyses were performed using GraphPad Prism 9.0. Two-way ANOVA followed by Dunnett’s multiple comparisons has been used. Quantitative PCR data were presented as the geometric mean with geometric standard deviation, while FC data were expressed as mean±standard deviation (SD). P-values below 0.05 were considered statistically significant.

## Results

### The apoptotic ratio of cells

**Fig 1:**
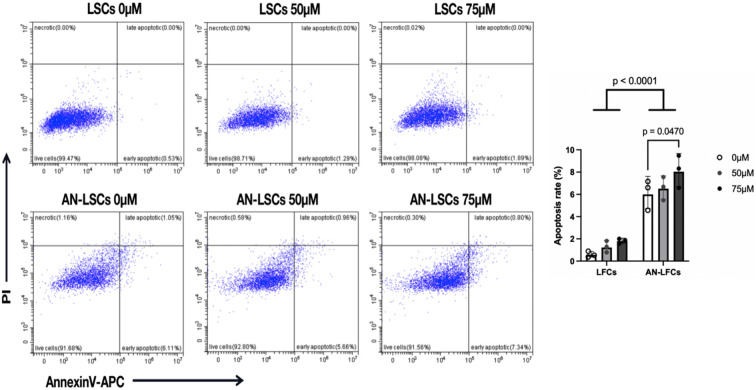
Apoptosis rates in limbal stromal cells (LSCs) and Aniridia-LSCs (AN-LSCs), after treatment with 0 µM, 50 µM and 75 µM Cobalt chloride (CoCl_2_). LSCs and AN-LSCs were treated with CoCl_2_ (0, 50 and 75 µM) for 48 h and stained with Annexin V-APC/PI before flow cytometry analysis. Data represent mean±SD from three independent experiments. Two-way ANOVA followed by Dunnett’s test was used, significant p values are indicated. Apoptosis rates were significantly higher in AN-LSCs compared to LSCs (p < 0.0001). Additionally, treatment with 75 µM CoCl_2_ significantly increased apoptosis in AN-LSCs compared to untreated controls (p = 0.0470).

Flow cytometry revealed a significantly higher apoptosis rate in AN-LSCs, than in LSCs (p < 0.0001). In AN-LSCs, 75 µM CoCl_2_ treatment significantly increased apoptosis rate, compared to untreated AN-LSCs (p = 0.0470) (Fig 1).

### mRNA levels of apoptosis-related markers

**Fig 2:**
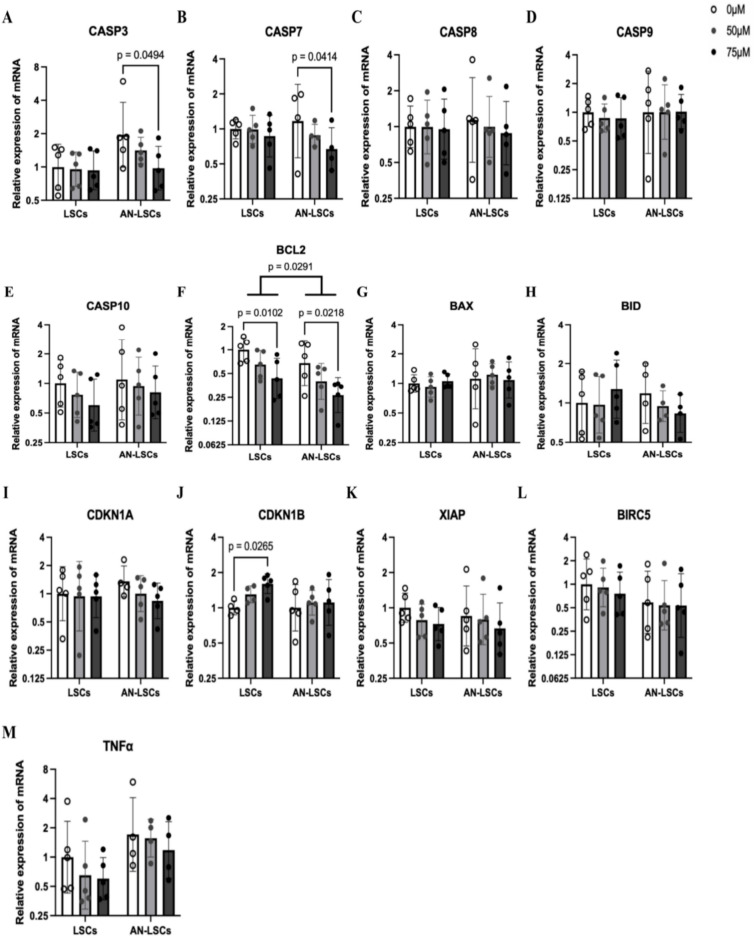
mRNA levels of CASP family markers (A-E), BCL2 family markers (F-H) and other apoptosis-related markers (I-M) in limbal stromal cells (LSCs) and in Aniridia-LSCs (AN-LSCs), after treatment with 0 µM, 50 µM and 75 µM Cobalt chloride (CoCl_2_). *CASP3, CASP7, CASP8, CASP9* and *CASP10* mRNA levels (A-E), *BCL2, BAX* and *BID* mRNA levels (F-H) and *CDKN1A, CDKN1B, TNFα, XIAP* and *BIRC5* mRNA levels (I-M) are shown. Values are expressed on a logarithmic scale (Log 2) with geometric mean±geometric standard deviation. Two-way ANOVA followed by Dunnett’s test was used, significant p values are indicated. *BCL2* mRNA levels were significantly higher in AN-LSCs compared to LSCs (p = 0.0291). In LSCs, *BCL2* mRNA level significantly decreased (p = 0.0102) and *CDKN1B* level significantly increased using 75 µM CoCl_2_ treatment. In addition, in AN-LFCs, 75 µM CoCl₂ treatment significantly decreased *CASP3, CASP9* and *BCL2* mRNA levels (p = 0.0494; p = 0.0414; p = 0.0218).

In AN-LSCs, *BCL2* mRNA levels were significantly downregulated, compared to LSCs (p = 0.0291). In LSCs, 75 µM CoCl_2_ treatment significantly downregulated *BCL2* mRNA levels (p = 0.0102) and upregulated *CNKN1B* mRNA levels (p = 0.0265). In AN-LSCs, 75 µM CoCl_2_ treatment downregulated *CASP3* (p = 0.0494), *CASP7* (p = 0.0414) and *BCL2* mRNA levels (p = 0.0218). Beyond these findings, there was no significant difference in the expression of other genes across different concentrations of CoCl_2_ treatment (Fig 2).

### Protein levels of apoptosis-related markers

**Fig 3:**
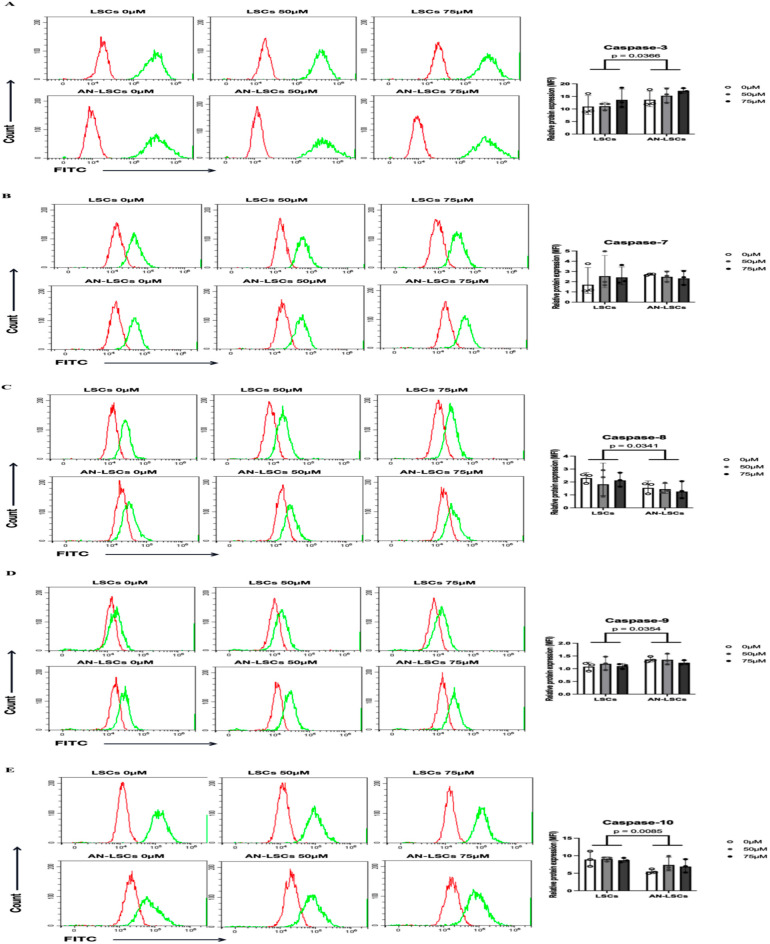
Proteins levels of Caspase family markers in limbal stromal cells (LSCs) and in Aniridia-LSCs (AN-LSCs), after treatment with 0 µM, 50 µM and 75 µM Cobalt chloride (CoCl_2_), using flow cytometry (A-E). Data represent mean±SD from three independent experiments. Two-way ANOVA followed by Dunnett’s test was used, significant p values are indicated. Protein levels of Caspase-3, Caspase-7, Caspase-8, Caspase-9, and Caspase-10 are shown. Representative histograms show primary antibody staining in green, while staining with the corresponding secondary antibody alone (negative control) is shown in red. MFI: mean fluorescence intensity, normalized to the secondary antibody control. In AN-LSCs, Caspase-3 and Caspase-9 protein levels were significantly higher and Caspase-8 and Caspase-10 protein levels were significantly lower than in LSCs (p≤0.0366). Nevertheless, there was no significant difference in protein levels of Caspase family members in any of the analysed subgroups, using different CoCl_2_ treatment.

**Fig 4:**
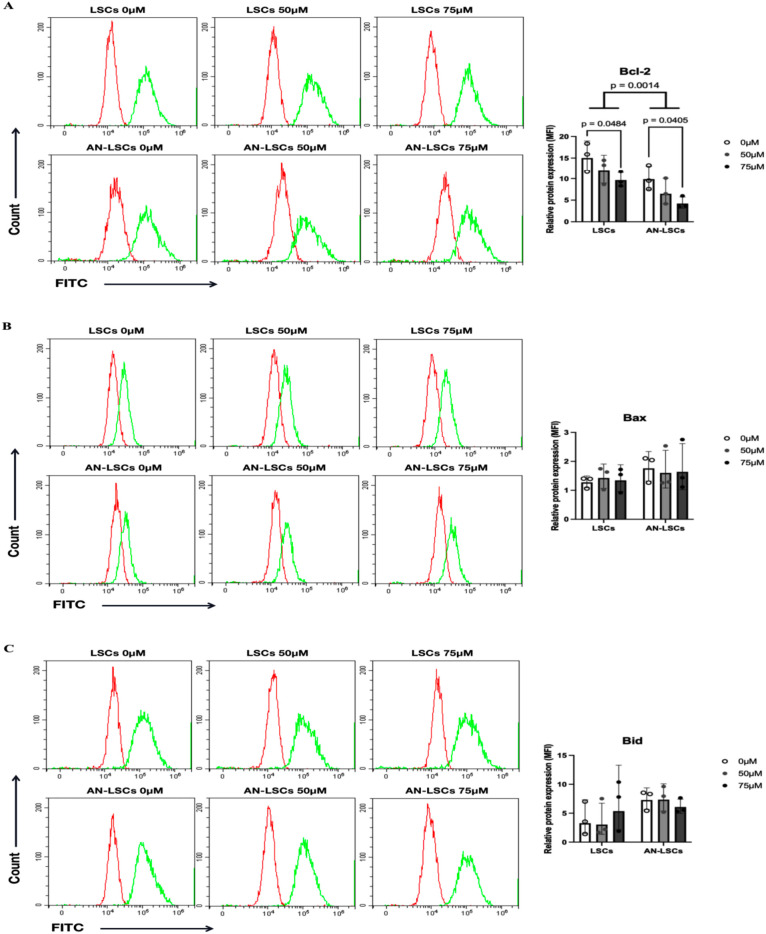
Protein levels of Bcl-2 family markers in limbal stromal cells (LSCs) and in Aniridia-LSCs (AN-LSCs), after treatment with 0 µM, 50 µM and 75 µM Cobalt chloride (CoCl_2_), using flow cytometry (A-C). Data represent mean±SD from three independent experiments. Two-way ANOVA followed by Dunnett’s test was used, significant p values are indicated. Bcl-2, Bax and Bid protein levels are shown. Representative histograms show primary antibody staining in green, while staining with the corresponding secondary antibody alone (negative control) is shown in red. MFI: mean fluorescence intensity, normalized to the secondary antibody control. Bcl-2 protein level was significantly lower in AN-LSCs, than in LSCs (p = 0.0014). In addition, 75 µM CoCl₂ treatment significantly decreased Bcl-2 protein level both in LSCs and AN-LSCs (p = 0.0484; p = 0.0405).

**Fig 5:**
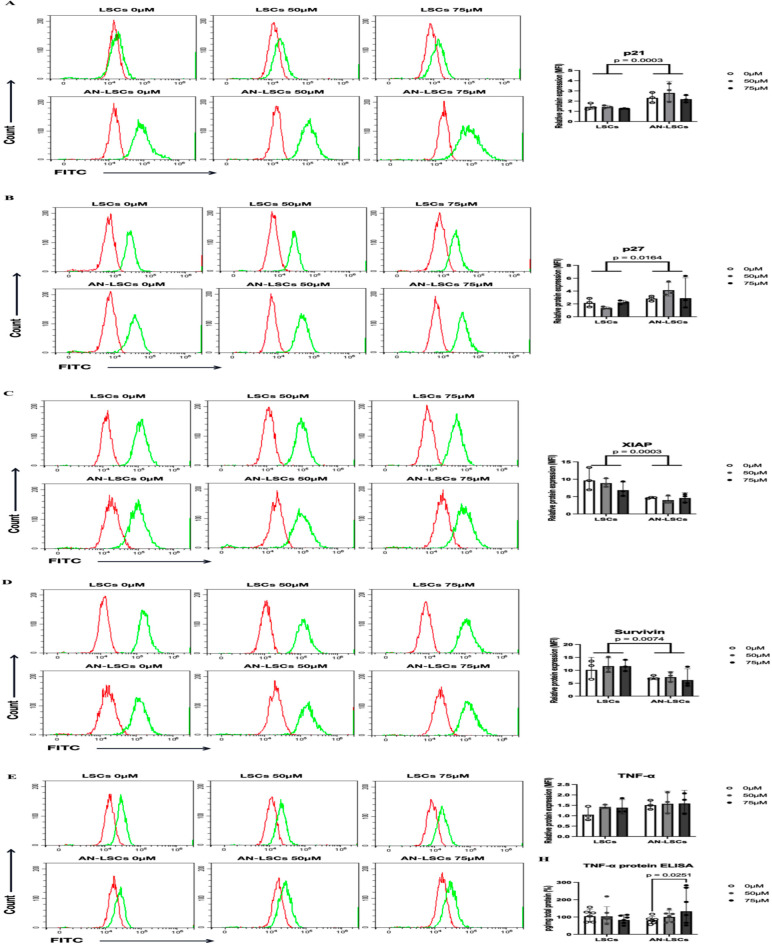
Protein levels of other apoptosis-related markers in limbal stromal cells (LSCs) and in Aniridia-LSCs (AN-LSCs), after treatment with 0 µM, 50 µM and 75 µM Cobalt chloride (CoCl_2_), using flow cytometry (A-E) and ELISA (H). Data represent mean±SD from three independent experiments. Two-way ANOVA followed by Dunnett’s test was used, significant p values are indicated. p21, p27, XIAP, TNF-α, and Survivin protein levels are shown. Representative histograms show primary antibody staining in green, while staining with the corresponding secondary antibody alone (negative control) is shown in red. MFI: mean fluorescence intensity, normalized to the secondary antibody control. In AN-LSCs, p21 and p27 protein levels were significantly higher (p = 0.0003; p = 0.0164) and XIAP and Survivin protein levels were significantly lower (p = 0.0003; p = 0.0074), than in LSCs, using flow cytometry. In addition, using ELISA, in cell culture supernatant of AN-LSCs, TNF-α protein levels were significantly higher following 75 µM CoCl_2_ treatment, than in untreated controls (p = 0.0251).

Compared to LSCs, the protein levels of Caspase-3 (p = 0.0366), Caspase-9 (p = 0.0354), p21 (p = 0.0003) and p27 (p = 0.0164) were significantly upregulated, while Caspase-8 (p = 0.0341), Caspase-10 (p = 0.00085), Bcl-2 (p = 0.0014), XIAP (p = 0.0003) and Survivin (p = 0.0074) were significantly downregulated in AN-LSCs (Figs 3–5).

In LSCs and in AN-LSCs, 75 µM CoCl_2_ treatment significantly downregulated Bcl-2 protein levels (p = 0.0484; p = 0.0405) (Fig 4). In the cell culture supernatant, TNF-α protein levels increased significantly (p = 0.0251) (Fig 5).

## Discussion

In this study, we systematically identified significant dysregulation of apoptosis in corneal limbal stromal cells from patients with congenital aniridia, along with their distinct response patterns to hypoxic stress. Firstly, our study revealed that AN-LSCs exhibit a significantly higher apoptotic rate than healthy LSCs, accompanied by a significant upregulation of pro-apoptotic proteins (Caspase-3/9, p21/p27) and a widespread downregulation of anti-apoptotic molecules (Bcl-2, XIAP, Survivin). Secondly, we observed that 75 µM CoCl_2_-induced hypoxic environment further exacerbated apoptosis in AN-LSCs, while LSCs exhibited only downregulation of BCL2 mRNA and protein levels. Notably, AN-LSCs showed unexpected downregulation of pro-apoptotic genes such as CASP 3/7 and BCL2 mRNA levels after hypoxia, suggesting that there may be a compensatory feedback mechanism for the regulation of their apoptotic pathway. Finally, we also found that the changes in mRNA and protein levels were not completely consistent, suggesting that post-transcriptional modification or protein stability may play an important role in the apoptotic regulation of limbal stromal cells in AAK. This could be observed regarding CASP3 mRNA downregulation in AN-LSCs after hypoxia, although its basal protein levels were significantly increased.

**Fig 6:**
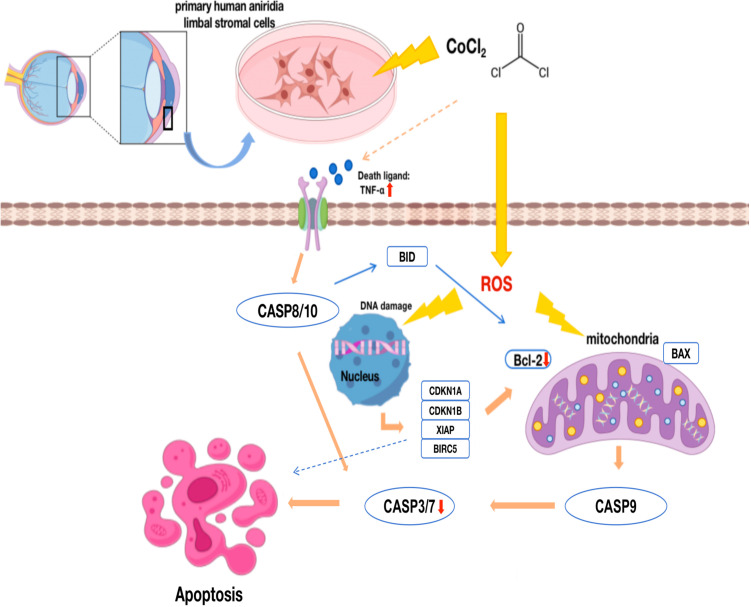
Schematic representation of the CoCl_2_-mediated apoptotic pathway in primary human aniridia limbal stromal cells (AN-LSCs). In AN-LSCs, CoCl_2_ induces a hypoxic environment and stimulates reactive oxygen species (ROS) production, which in turn affects mitochondrial function, causes DNA damage, and triggers apoptosis. This is accompanied by differential expression of related genes, including downregulation of the anti-apoptotic *Bcl-2* and the pro-apoptotic *CASP3* and *CASP7*. CoCl_2_ also increases extracellular TNFα levels and activates the extrinsic apoptotic pathway, further promoting apoptosis.

Previous studies have shown that dysfunction or depletion of corneal limbal epithelial stem cells and limbal stromal cells leads to impaired renewal of the corneal epithelium, resulting in a range of ocular surface disorders [23,24]. The significant downregulation of Bcl-2 and XIAP, accompanied by the activation of Caspase-3 and 9, may contribute to the depletion of corneal limbal stromal cells via mitochondria-dependent apoptotic pathways in AN-LSCs [25]. Interestingly, in AN-LSCs, CoCl_2_ treatment suppressed some pro-apoptotic genes (CASP3/7 and BCL2 mRNA) although it further elevated the apoptosis rate. This paradoxical phenomenon may result from negative feedback regulatory mechanisms activated in cells undergoing prolonged apoptosis, such as cell cycle arrest mediated by p21/p27, which serves to limit further amplification of apoptotic signaling [26,27]. Liu et al. reported in their study on herpetic eye disease that inflammation can cause significant damage to the corneal limbal microenvironment [28]. In the present study, the marked increase in TNF-α protein levels in the supernatant of AN-LSCs following hypoxic stress suggests that the inflammatory microenvironment exacerbated changes through the extrinsic apoptotic pathway involving Caspase-8 and 10. These findings provide experimental support for the hypothesis that inflammation-induced apoptosis contributes to the pathogenesis of AAK (Fig 6).

In corneal epithelial cells, Caspase-3 expression is closely associated with inflammation and cell death [29]. For example, in dry eye disease models, Caspase-3 has been shown to exacerbate disease progression by promoting pyroptosis, a form of programmed cell death in corneal epithelial cells [30]. Similarly, elevated Caspase-3 expression has been observed in corneal alkali burns, where it contributes to inflammation and tissue damage. Notably, inhibition of Caspase-3 in these models reduced both inflammation and corneal damage, thereby promoting tissue healing [31]. Further evidence of Caspase-3’s role in corneal pathology comes from studies showing its upregulation in response to oxidative stress, again linking it to apoptosis in corneal epithelial cells [31]. Its expression is also markedly increased in viral keratitis, suggesting a central role in corneal injury caused by viral infections [32]. Beyond its apoptotic functions, Caspase-3 also modulates corneal immune responses and tissue repair, indicating its broader significance in inflammation-related corneal damage [33].

Nevertheless, the role of Caspase-3 in limbal stromal cells has not been analysed, yet. The present study confirmed elevated Caspase-3 expression in aniridia-derived limbal stromal cells *in vitro*, in a congenital hereditary eye disease model [30]. In addition, in contrast to diabetic keratopathy models, AN-LSCs exhibited significant Bcl-2 depletion even under normoxic conditions, suggesting that genetic defects may predispose these cells to a lower apoptotic threshold, independent of environmental triggers [34]. Importantly, this study is the first to report abnormal downregulation of pro-apoptotic genes in AN-LSCs under hypoxic stress—a phenomenon not previously described in other corneal disease models. This unique response may point to AAK-specific mechanisms of apoptosis regulation at mRNA and protein levels and offer novel insights into potential therapeutic targets in AAK.

To address impaired corneal epithelial regeneration in patients with congenital aniridia, various therapeutic strategies are under investigation, including limbal stem cell transplantation and gene therapy. These approaches aim to restore or replace damaged stem cell function, thereby promoting normal corneal epithelial renewal [35,36]. However, their clinical application remains challenging due to issues such as limited survival of transplanted cells, immune rejection, and uncertain long-term efficacy [23,35]. In terms of clinical translational value, our findings suggest that elevated Caspase 3 protein levels, in combination with dual downregulation of Bcl-2 at both mRNA and protein levels, may serve as a potential biomarker set for early AAK diagnosis. Therapeutically, Caspase inhibitors (e.g., z-VAD-fmk) and Bcl-2 analogues (e.g., ABT-199) may help restore AN-LSC viability [37,38]. Additionally, blocking TNFα signaling (e.g., with etanercept) may help interrupt the vicious cycle of hypoxia, inflammation, and apoptosis [39]. The observed upregulation of p21 and p27 further suggests that CDK inhibitors may provide a novel approach by modulating cell cycle progression in the limbal niche.

While this study yielded important findings, it also has limitations. First, the *in vitro* CoCl_2_ model does not fully replicate the *in vivo* hypoxic microenvironment. Second, the limited sample size may affect statistical power. Moreover, the specific regulatory mechanisms behind the discrepancies between mRNA and protein expression such as miRNA-mediated translational repression remain to be elucidated. In future research, we plan to expand upon the CoCl_2_ data using alternative hypoxia-induced oxidative stress models, and to explore the functional reversal of the apoptotic phenotype by repairing the Bcl-2 gene in AN-LSCs using CRISPR/Cas9 technology. Future studies should aim to increase sample sizes, integrate functional assays, and utilize in vivo models to more effectively translate our findings into clinical applications.

## Conclusions

The present study confirms the activation of apoptotic pathways in corneal limbal stromal cells in AAK. Specifically, the combined effect of mitochondria-dependent apoptosis and suppression of anti-apoptotic molecules contributes to impaired corneal regeneration. Hypoxic stress further exacerbates this pathological process, in part through the synergistic influence of inflammatory mediators such as TNFα. These findings suggest that targeted modulation of the Bcl-2/Caspase axis or intervention in the hypoxia-inflammation-apoptosis cascade may represent promising therapeutic strategies to restore corneal homeostasis in patients with AAK.
